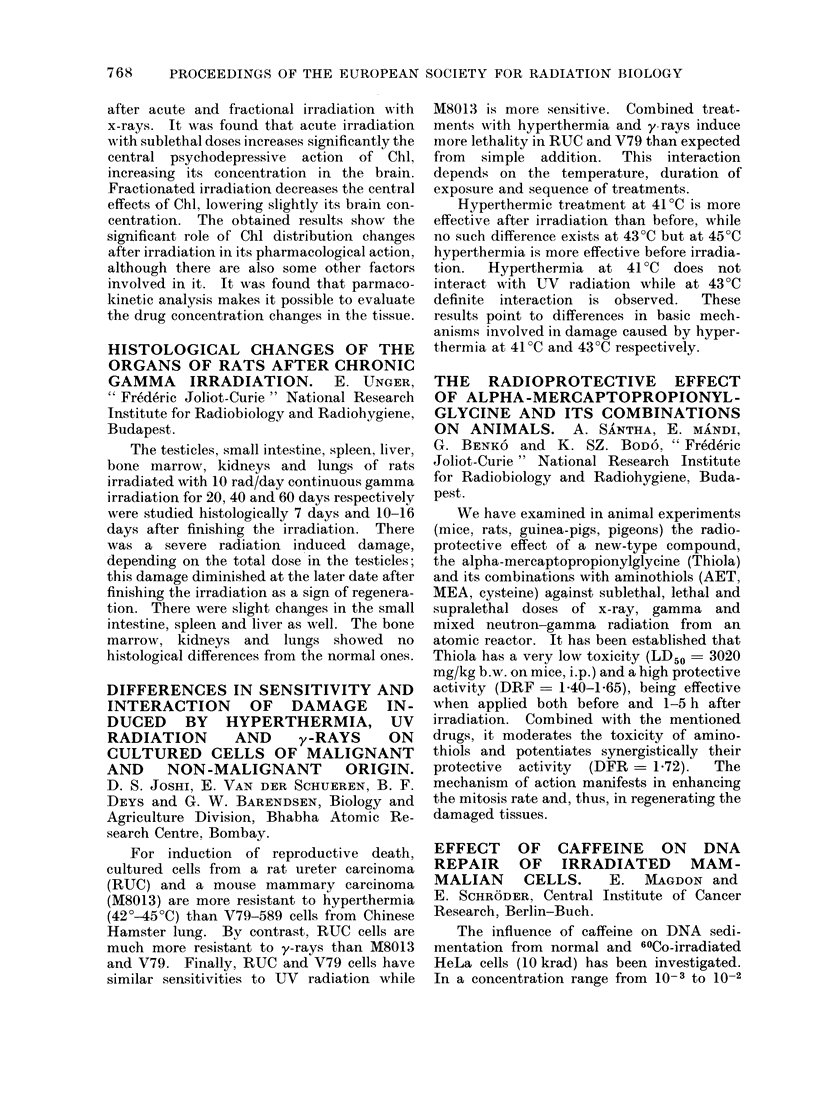# Proceedings: The radioprotective effect of alpha-mercaptopropionylglycine and its combinations on animals.

**DOI:** 10.1038/bjc.1975.347

**Published:** 1975-12

**Authors:** A. Sántha, E. Mándi, G. Benkó, S. Z. Bodó


**THE RADIOPROTECTIVE EFFECT OF ALPHA-MERCAPTOPROPIONYLGLYCINE AND ITS COMBINATIONS ON ANIMALS.** A. Sántha, E. Mándi, G. Benkó and K. Sz. Bodó, "Fréderic Joliot-Curie" National Research Institute for Radiobiology and Radiohygiene, Budapest.

We have examined in animal experiments (mice, rats, guinea-pigs, pigeons) the radioprotective effect of a new-type compound, the alpha-mercaptopropionylglycine (Thiola) and its combinations with aminothiols (AET, MEA, cysteine) against sublethal, lethal and supralethal doses of x-ray, gamma and mixed neutron–gamma radiation from an atomic reactor. It has been established that Thiola has a very low toxicity ($LD_{50} = 3020$ mg/kg b.w. on mice, i.p.) and a high protective activity (DRF = 1·40–1·65), being effective when applied both before and 1–5 h after irradiation. Combined with the mentioned drugs, it moderates the toxicity of aminothiols and potentiates synergistically their protective activity (DFR = 1·72). The mechanism of action manifests in enhancing the mitosis rate and, thus, in regenerating the damaged tissues.